# Novel Insights into the Regulatory Role of Nuclear Factor (Erythroid-Derived 2)-Like 2 in Oxidative Stress and Inflammation of Human Fetal Membranes

**DOI:** 10.3390/ijms21176139

**Published:** 2020-08-26

**Authors:** Ramkumar Menon, Morgan R Peltier

**Affiliations:** 1 Division of Maternal-Fetal Medicine and Perinatal Research, Department of Obstetrics and Gynecology, The University of Texas Medical Branch at Galveston, Galveston, TX 77555, USA; 2 Department of Foundations of Medicine, New York University-Long Island School of Medicine, Mineola, NY 11501, USA; Morgan.Peltier@nyulangone.org; 3 Department of Obstetrics and Gynecology, New York University-Long Island School of Medicine, Mineola, NY 11501, USA

**Keywords:** antioxidant, sulforaphane, coffee, green vegetables, preterm birth, nutrition, PPARγ

## Abstract

Fetal membrane dysfunction in response to oxidative stress (OS) is associated with adverse pregnancy outcomes. Nuclear factor (erythroid-derived 2)-like 2 (Nrf2) is one of the regulators of innate OS response. This study evaluated changes in Nrf2 expression and its downstream targets heme oxygenase (HO-1) and peroxisome proliferator-activated receptor gamma (PPARγ) in fetal membranes during OS and infection in vitro. Furthermore, we tested the roles of sulforaphane (SFN; an extract from cruciferous vegetables) and trigonelline (TRN; an aromatic compound in coffee) in regulating Nrf2 and its targets. Fetal membranes (*n* = 6) collected at term were placed in an organ explant system were treated with water-soluble cigarette smoke extract (CSE), an OS inducer (1:10), and lipopolysaccharide (LPS; 100 ng/mL). Nrf2 expression, expression, its enhancement by sulforaphane (SFN, 10 µM/mL) and down regulation by TRN (10uM/mL) was determined by western blots. Expression of Nrf2 response elements PPARγ (western) heme oxygenase (HO-1), and IL-6 were quantified by ELISA. CSE and LPS treatment of fetal membranes increased nrf2, but reduced HO-1 and PPARγ and increased IL-6. Co-treatment of SFN, but not with TRN, with CSE and LPS increased Nrf2 substantially, as well as increased HO-1 and PPARγ and reduced IL-6 expression. Risk factor-induced Nrf2 increase is insufficient to generate an antioxidant response in fetal membranes. Sulforaphane may enhance innate antioxidant and anti-inflammatory capacity by increasing NRF-2 expression.

## 1. Introduction

The World Health Organization recently estimated the global preterm birth rate for singleton gestation at 10.5% [1]. The preterm birth rate has increased in the United States by as much as 30% during the last 25 years despite advances in medical care [2]. The most common phenotype of preterm birth is spontaneous preterm birth (PTB) of unknown etiology. Approximately 60% PTB are spontaneous, and 30–40% of these are preceded by preterm prelabor rupture of the fetal membranes (pPROM) [2,3,4]. Approximately 50% of PTB and 70% of pPROM are associated with microbial invasion of the amniotic cavity (MIAC) and intraamniotic inflammation (IAI) [5,6,7]. Inflammatory changes that precede PTB, such as leukocyte activation, increased inflammatory cytokines and chemokines, and collagenolysis of the extracellular matrix by metalloproteinases (MMPs), resulting in loss of membrane structural integrity, myometrial activation, and cervical ripening, are well documented by experimental and clinical studies [8,9,10,11]. We have reported the heterogeneity in the inflammatory response (cytokines/chemokines, toll-like-receptors, and their interactions) associated with IAI and PTB risk factors [12,13,14].

Recent literature has indicated oxidative stress (OS) and the OS-associated damage caused by the generation of reactive oxygen species (ROS), an inseparable component of inflammation, can also contribute to PTB and pPROM pathology-even in the absence of infection [15,16]. A healthy pregnancy is characterized by a stable balance between ROS and antioxidants [15,17,18,19]. An imbalance in the redox status is a pathologic feature underlying many pregnancy complications [20,21,22,23]. Risk factors associated with PTB and pPROM can generate superoxide, hydrogen peroxide, hydroxyl ions, and nitric oxide that can damage collagen matrix and consume antioxidant defenses. These events can trigger uterine contractions (labor), leading to PTB [24,25].

Clinical trials using supplements that reduce OS and improve pregnancy outcome have had minimal success [26,27,28,29,30,31]. Antioxidant therapy has also not been successful for PTB and pPROM prevention, and there is little evidence to justify their clinical use for these indications [27]. Antioxidants typically target an OS pathway or a specific free radical that may vary among OS risk factors like infection, smoking, poor nutrition or obesity. The approach of administering antioxidants after diagnosis of preterm labor or pPROM is unlikely to delay delivery unless it can stop pathophysiologic pathways of labor induced by OS damage [24]. This suggests that there is a need for a better understanding of the natural response to OS and how its manipulation can affect pregnancy outcome [32,33,34,35,36,37].

Nuclear factor (erythroid-derived 2)-like 2 (Nrf2) is a leucine zipper transcription factor that regulates multitudes of antioxidant responses in mammalian cells. Nrf2 maintains intracellular homeostasis in response to ROS through the generation of antioxidants hemeoxygenase-1 (HO-1) and carbon monoxide (CO) in conjunction with anti-inflammatory nuclear receptors like peroxisome proliferator-activated receptor gamma (PPARγ) [31,32,33,34,35,36,37,38,39,40]. Normally, Nrf2 is maintained in the cytoplasm bound tightly to Kelch-like ECH-associated protein 1(keap-1) [41,42,43,44]. ROS causes Nrf2 to dissociate from keap-1, translocate to the nucleus, and transcribe genes that help restore oxidative balance [32,35,42,43,44,45,46]. Nrf2 null mice show impaired induction of these antioxidant defenses and greater oxidative damage and emphysema after exposure to cigarette smoke [38]. Activation of Nrf2-regulated pathways may be an important part of the natural response to environmental exposures that maintain pregnancy through induction of hemeoxygenase-1 that is upregulated by Nrf2 and makes carbon monoxide (CO), a gas with potent anti-inflammatory properties. Administration of CO significantly reduced adverse outcomes in a mouse model of infection-mediated PTB [39,40]. In many cases, OS is overwhelming, and nrf2 increase is insufficient to produce redox imbalance. We postulate that substantial enhancement of Nrf2 and its downstream targets HO-1 and anti-inflammatory signaler, PPARγ, may be a better approach to improve pregnancy outcome.

Dietary changes may be the best approach to manipulation of Nrf2 expression because they are generally safe enough to give to all pregnant women and would not require identifying women at elevated risk for pPROM and preterm birth. One compound, Sulforaphane (SFN), has been widely investigated in this capacity as an anti-inflammatory mediator. It is an isothiocyanate released from cruciferous vegetables (broccoli, cabbages, and brussels sprouts) upon digestion and is a potent booster of the Nrf2 response in the cell [41,42,43]. Trigonelline (TRN) an alkaloid present in coffee and green coffee beans, has also been reported to affect Nrf2 expression. During the roasting process of coffee beans, trigonelline changes into N-methylpyridinium and nicotinic acid as its major products, which makes it a useful index of the degree of roasting [44,45,46] TRN, however, has been reported to reduce Nrf2 expression and activation. Numerous studies have examined the potential association of high coffee consumption with increased risk for PTB [47], but results have been confliction perhaps. This may be due to their focus on total caffeine consumption and reliance on food frequency questionnaires [48,49,50,51]. We hypothesize that SFN and TRN have opposite effects on the innate antioxidant/anti-inflammatory environment to help fetal growth or in response to OS and inflammation-inducing risks during pregnancy. To test this, we used human fetal membrane explant cultures and tested the role of SFN and TRN in regulating endogenous antioxidant response mediated through Nrf2.

## 2. Results

Western blots (Nrf2 and PPARγ) and ELISA (HO-1 and IL-6) were used to determine changes in expression and or production of various analytes in response to treatments with CSE and LPS either alone or in combination with SFN and TRN. Western blot data are presented as arbitrary units, and ELISA data are reported as ng/mL (HO-1) or pg/mL (IL-6 and IL-8). All experiments were performed using fetal membrane tissues from 6 different subjects.

### 2.1. CSE and LPS Increase Nrf2 Expressions in Human Fetal Membranes

Western blot analysis was used to determine the effect of CSE and LPS on Nrf2 expression in human fetal membranes tissue explants in culture. Nuclear fractions were used for determining Nrf2 expression. Both LPS and CSE increased Nrf2 expression after 6 h of exposure. Nrf2 expression was significantly higher after CSE treatment (1.018 ± 0.5627) compared to untreated controls maintained in the same tissue culture conditions (0.3604 ± 0.2267; *p* < 0.05). Although increased, significance was not reached after LPS treatment (0.5311 ± 0.2663) compared to control.

### 2.2. SFN Co-Treatment Augments CSE and LPS Response and Increase Nrf2

**Figure 1:**
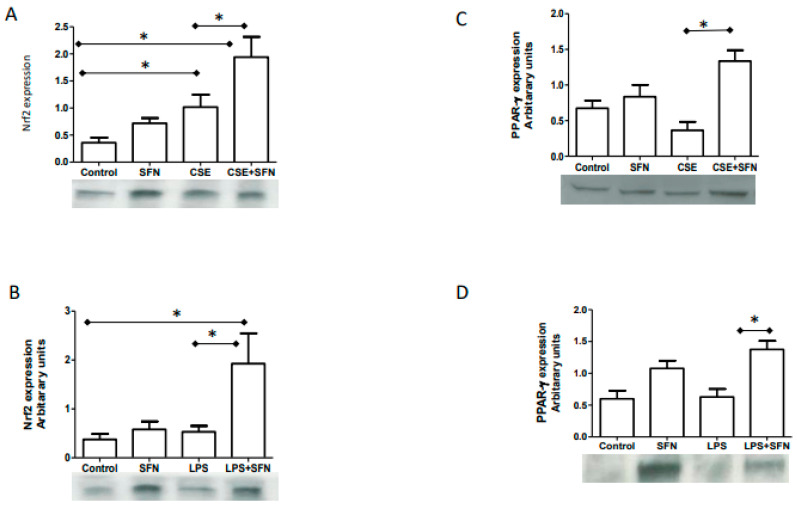
Representative western blots of Nrf2 (100kDa) and PPARγ (53 kDa) expressions in fetal membranes and graphical representation of data (*n* = 6) after 6 h of treatment with various factors.; * *p* < 0.05. (**A**): Fetal membranes treated with sulforaphane (SFN), cigarette smoke extract (CSE) and CSE+SFN. (**B**): Fetal membranes treated with SFN, lipopolysaccharide (LPS) and LPS+SFN. (**C**): Fetal membranes treated with SFN, CSE and CSE+SFN. (**D**): Fetal membranes treated with SFN, LPS and LPS+SFN.

We next examined augmentation of Nrf2 expression by SFN treatment. As shown in Figure 1A, co-treatment of SFN with CSE significantly increased Nrf2 expression in the fetal membrane cells (SFN+CSE—1.941 ± 0.9195; CSE—1.018 ± 0.5627; *p* < 0.05). SFN alone did not change Nrf2 expression in tissue explants compared to controls (0.7187 ± 0.2383 vs. 0.3604 ± 0.2267). Similarly, co-treatment of SFN with LPS significantly increased Nrf2 expression (1.927 ± 1.386) compared to either LPS (0.5311 ± 0.2663), SFN alone (0.5807 ± 0.3667 or control (0.3763 ± 0.2497) (*p* < 0.05 for all) (Figure 1B).

### 2.3. SFN Co-Treatment with CSE or LPS Increases PPARγ Expression

PPARγ is a downstream responder and activator of an anti-inflammatory response in the cell. We tested the effect of SFN on PPARγ expression by western blot in cytosolic fractions. As shown in Figure 1C, CSE produced an approximately 50% reduction in PPARγ expression (0.3645 ± 0.2892) compared to control (0.6738 ± 0.2643) although this reduction was statistically not significant. Co-treatment with SFN produced a four-fold increase in PPARγ expression (1.336 ± 0.3745; *p* < 0.001) compared to CSE. Similarly, LPS did not cause any change in PPARγ expression (0.6294 ± 0.2779; *p* = ns). However, PPARγ expression was significantly increased after co-treatment with SFN (1.376 ± 0.2974; *p* < 0.05) (Figure 1D).

### 2.4. TRN Co-Treatment with CSE and LPS Did Not Change Nrf2 and PPARγ Expressions

**Figure 2:**
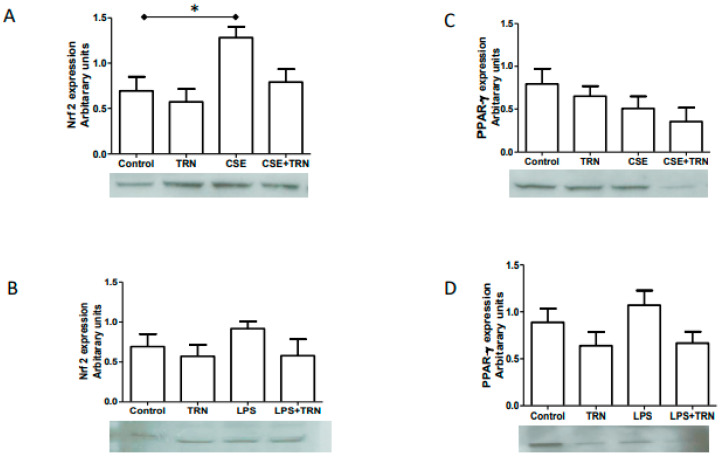
Representative western blots of Nrf2 (100 kDa) and PPARγ (53 kDa) expressions in fetal membranes and its graphical representation of data (*n* = 6) after 6 h of treatment with various factors; * *p* < 0.05. (**A**): Fetal membranes treated with trigonelline (TRN), cigarette smoke extract (CSE) and CSE+TRN. (**B**): Fetal membranes treated with trigonelline (TRN), lipopolysaccharide (LPS) and LPS+TRN. (**C**): Fetal membranes treated with TRN, CSE and CSE+TRN. (**D**): Fetal membranes treated with TRN, LPS and LPS+TRN.

NRF2 and PPARγ expressions were evaluated after co-treatment with TRN. Co-treatment with CSE did not result in a significant change in Nrf2 expression in fetal membrane explants (Figure 2A). Similarly, LPS+TRN also did not change Nrf2 expression levels (Figure 2B). Similar results were obtained for PPARγ where no significant difference was observed whether TRN or alone or when co-treated with either CSE or LPS (Figure 2C,D).

### 2.5. Endogenous HO-1 Levels are Increased by SFN Co-Treatment of CSE and LPS

**Figure 3:**
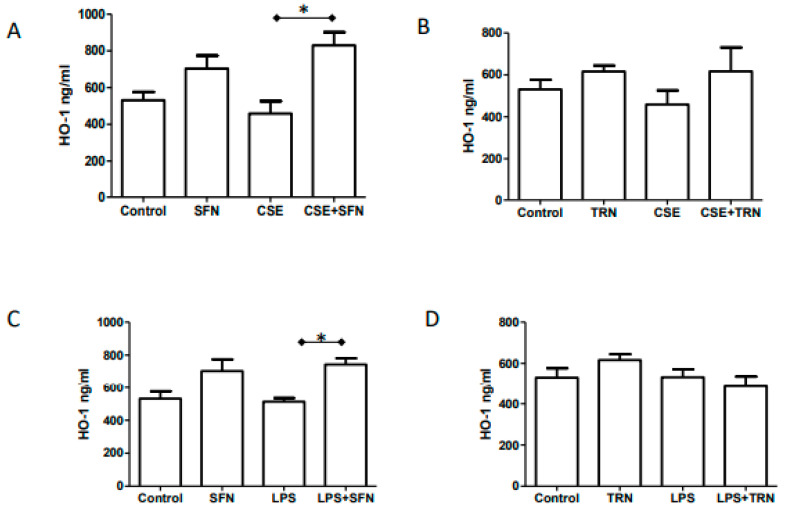
HO-1 levels in fetal membrane tissue homogenates determined by ELISA. * *p* < 0.05. (**A**): Fetal membranes treated with sulforaphane (SFN), cigarette smoke extract (CSE) and CSE+SFN. (**B**): Fetal membranes treated with SFN, lipopolysaccharide (LPS) and LPS+SFN. (**C**): Fetal membranes treated with trigonelline (TRN), CSE and CSE+TRN. (**D**)**.** Fetal membranes treated with TRN, LPS and LPS+TRN.

To determine the effect of Nrf2 increase associated changes in the OS response elements, we measured tissue levels of HO-1. As shown in Figure 3A, reduction of HO-1 after CSE treatment was not significant compared to control (458.7 ± 151.2 vs. 531.3 ± 100.7); however, co-treatment of CSE with SFN significantly increased HO-1 levels (830.6 ± 160.5; *p* < 0.01). We also noticed that SFN alone increased HO-1 (703.7 ± 157.9) compared to either control or CSE, but results did not reach statistical significance. This could be due to sample size. Similarly, LPS (along with SFN) significantly increased HO-1 level (743.7 ± 83.97 vs 512.8 ± 51.20; *p* < 0.05) in fetal membranes. TRN treatment had no effect on HO-1 levels when co-treated with either CSE or LPS (Figure 3C,D).

### 2.6. SFN Reduces IL-6 Release Induced by Both CSE and LPS

**Figure 4:**
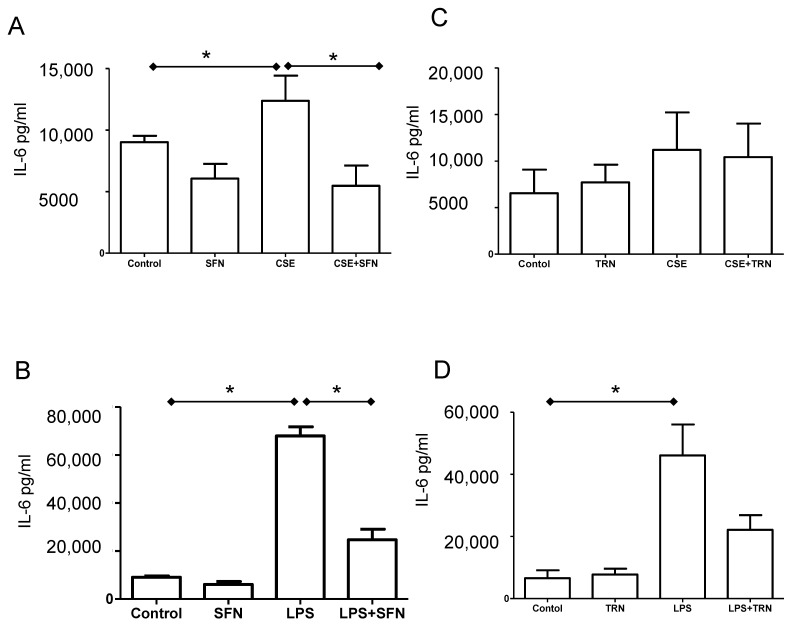
Interleukin (IL)-6 levels in culture supernatant from fetal membrane tissue homogenates determined by ELISA. * *p* < 0.05. (**A**): Fetal membranes treated with sulforaphane (SFN), cigarette smoke extract (CSE) and CSE+SFN. (**B**): Fetal membranes treated with SFN, lipopolysaccharide (LPS) and LPS+SFN. (**C**): Fetal membranes treated with trigonelline (TRN), CSE and CSE+TRN. (**D**). Fetal membranes treated with TRN, LPS and LPS+TRN.

As expected and confirming our prior studies, both CSE and LPS treatments increased the release of IL-6 (CSE-12379 ± 4594; LPS- 67918 ± 7513; *p* < 0.05 for both) from fetal membrane explants compared to controls (9016 ± 1164). Co-treatment of SFN with CSE and LPS reduced IL-6 to 5479 ± 3664 and 24613 ± 8890, respectively; both *p* < 0.05) (Figure 4A,B). TRN treatment had no effect on either CSE induced IL-6 production (Figure 4C); however, although results did not reach statistical significane, significant, co-treatment with LPS showed a 50% reduction in IL-6 (Figure 4D).

## 3. Discussion

**Figure 5:**
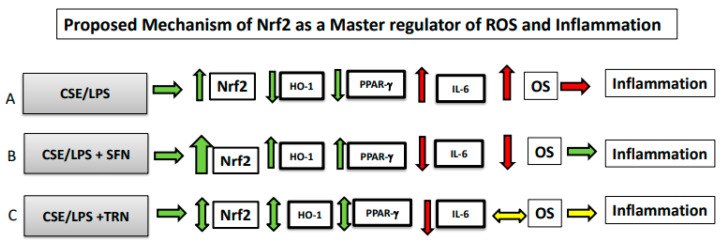
**Summary of data:** A balanced oxidative stress (OS) and inflammation work cooperatively during gestation to effectively remodel tissues and maintain pregnancy. Sterile (non-infectious) and infectious risk factors can override this balanced activity and cause increased OS and inflammation to cause adverse pregnancy outcomes such as PTB or pPROM. **↓, down regulation;** **↑, upregulation;** **↔, no change.** (**A**): Oxidative stress (OS) inducer CSE and LPS can increase or do not change Nrf2 expression in fetal membranes. Stimulations; however, result in decreased antioxidant HO-1 and anti-inflammatory PPARγ levels and an increase in IL-6. In summary, CSE and LPS increases OS and inflammation in fetal membranes. (**B**). Co-treatment with sulforaphane (SFN) leads to a substantial increase in Nrf2 (thick up arrow) and subsequent increase in HO-1, PPARγ and decrease in IL-6 suggesting an overall decrease in OS leading to inflammation in fetal membranes. (**C**). Co-treatment with TRN leads to no change in Nrf2, HO-1, PPARγ and IL-6 suggesting that no impact on overall OS and inflammation (yellow arrow) in fetal membranes. This model depicts changes observed in this study, and it may not indicate a linear relationship between tested markers in response to stimulants used.

In this study, we evaluated the effects of Nrf2 activator, Sulforaphane (SFN) and inhibitor, Trigonelline (TRN), on Nrf2-driven antioxidant pathways in human fetal membrane explants exposed to oxidative stress by infection or cigarette smoke components. Principle findings of our studies are: (1) OS inducer, CSE, and proxy for infection, LPS, increased Nrf2 expression and IL-6 production by fetal membranes but decreased intracellular levels of PPARγ and HO-1; (2) SFN alone had no effect on Nrf2 expression; (3) Co-treatment of SFN with both CSE and LPS increased Nrf2, PPARγ, HO-1 and reduced IL-6; and (4) TRN had no detectible effects on fetal membrane Nrf2 expression or its downstream targets. This suggests that SFN can partly minimize OS and inflammation in fetal membranes and improve the overall antioxidant capacity of fetal membranes and that the pathway is refractory to modulation by TRN. These findings are summarized in Figure 5.

Nrf2 is a critical factor that provides a multi-level response to OS in a cell. Under basal conditions, Nrf2 is sequestered in the cytoplasm, bound to the Kelch-like ECH-associated protein 1 (Keap1) in an inactive state. Upon exposure to ROS, it becomes activated and translocate to the nucleus where it binds to specific gene promoters of the antioxidant response elements (ARE) that include phase II detoxifying enzymes like quinone oxidoreductase and antioxidant enzymes [52,53]. HO-1 is one of the key genes that is transcribed by Nrf2 binding [54,55]. Nrf2-mediated HO-1 induction decreases OS damage induced by various pro-oxidants, catalyzing the degradation of heme to biliverdin, iron, and carbon monoxide (CO), all of which exert anti-inflammatory and antioxidant functions [55,56]. Mice deficient in HO-1 have increased rates of fetal wastage, low birth weight and preeclampsia—some of which can be alleviated by the administration of CO [57].

PPARs are ligand-activated transcription factors. PPARs function as a heterodimer in association with co-activator complex that binds to the promoter region of specific genes that contain DNA sequence termed peroxisome proliferators response elements (PPREs) [58]. This binding can cause activation or down regulation of various genes. The role of PPARγ in fetal membrane anti-inflammatory response has been well reported [59,60]. Lappas et al. have shown that the down regulation of PPARγ and activation of NF-κB is a pathway for inflammatory activation in reproductive tissues preparing them for parturition [61,62]. Activators of PPARγ, including Sulfasalazine and PGJ2, have been shown to prevent preterm birth in animal models [63]. Therefore, the regulation of PPARγ is critical for controlling inflammation that contributes to PTB and pPROM. IL-6 is well-established biomarker of infection and inflammation during pregnancy. Although it is not a good indicator of any exposure risk or pathology, it likely plays roles in neurodevelopment disorders as offspring of mice that received a single injection of IL-6 during pregnancy have autistic-like behaviors. Both CSE and LPS increased its production, and SFN co-treatment reduced IL-6—indicating downregulation of an overall inflammatory process. This suggests that SFN may help reduce the consequences of exposure to inducers of OS such as smoking and infection.

Differential expression of Nrf2 in human fetal membranes was first reported by Martha Lappas’ group [64] where they reported decreased Nrf2 mRNA and nuclear protein expressions after term labor and delivery but no change in PTB. Silencing of Nrf2 was associated with increased expression of inflammatory cytokines in primary amnion cells [64]. In this report, labor (a condition associated with OS) showed a decrease in Nrf2, whereas in our in vitro explant models (term not in labor) Nrf2 was slightly increased in response to CSE-induced OS. As shown, this effect was insufficient to minimize OS or inflammation without SFN supplementation. Although the net effect—increase in inflammation and OS—was similar in both studies, OS at term labor, massive levels of senescence and other endocrine and paracrine factors not represented in our in vitro model may contributing to an overall decrease in Nrf2. This could be a limitation of in vitro models. Cellular level differences (amnion, chorion, and mesenchymal cells), if any, is also not investigated in this study, and we present an overall tissue level change. However, a study by Chigusa et al. using amnion mesenchymal cell has shown activation of Nrf2 inhibited thrombin-induced inflammatory mediator release suggesting that cellular components provide supporting evidence to what we have observed at the tissue level [65].

A recent study by Zhang et al. examined the role of Keap-1/Nrf2 signaling pathway activation by OS in membranes from preterm birth following preterm PROM and rupture of membranes at term [66]. This study reported increased ROS levels and decreased antioxidant enzymes in both pPROM and term spontaneous rupture groups that were mechanistically associated with Nrf2/Kaep mediated signaling [66]. Feng et al. reported higher levels of Nrf2 and HO-1 levels in placental specimens from preeclampsia [56]. Higher levels of Nrf2 and HO-1 levels was also associated with increased OS in placenta. Our studies confirm these reports, and we provide additional data to show that supplementation of Nrf2 enhancers—such as dietary supplements like SFN or consuming cruciferous vegetables diet during pregnancy, may enhance endogenous Nrf2 levels to minimize OS and inflammation. Sulforaphane can also interact with Nrf2/Keap1 complex and prevent the ubiquitination of Nrf2 by modification of cysteine residues in Keap1. McMahon et al. have reported that sulforaphane blocks proteasome-mediated degradation and stabilizes Nrf2 [67,68].

OS occurs when the balance between ROS and antioxidant levels is disrupted to favor excessive ROS [69]. OS can produce a wide spectrum of genetic, metabolic, and cellular consequences because ROS can have detrimental effects on lipids, proteins and nucleic acids, disrupting their expression, production structure, and function [16,17,70,71]. The resulting damage leads to activation of various pathways that can determine cell fate like senescence, apoptosis and necrosis [24,72]. Clinical trials are often conducted under the assumption that OS is an underlying pathology and that simply increasing antioxidants will improve the outcome. More likely, it is an inability for the body to respond to OS caused by various sources that are contributing to adverse pregnancy outcomes. A better understanding of OS-related responses and the molecules controlling OS-related events that result in adverse pregnancy outcome may help design better clinical trials and therapies that can address 1) OS-inducing risk factors, 2) types of OS response, and 3) exploitation of the body’s natural mechanisms for controlling OS. Regulation of OS is essential to restrict inflammatory uterotonic pathways, and identification of the regulatory center for OS response is even more important. To note, changes observed in the expression and or release of markers in response to treatments provide support association between pro or antioxidant properties and Nrf2, but they are not mechanistically proven in our study.

Regulation of inflammation and OS contributing to PTB and pPROM pathways require enhancement of overall antioxidant and anti-inflammatory status of the intrauterine tissues. Consumption easily available cruciferous vegetables, a rich source of SFN, to enhance cellular antioxidant capacities can be considered as a simple, inexpensive, and adequate approach to maintain redox balance. SFN supplements are already sold over the counter as a nutritional supplement and could be administered the same way prenatal vitamins are now to reduce the risk of some adverse pregnancy outcomes. Although we hypothesized that TRN, an ingredient of coffee, would aggravate inflammatory response and reduce the antioxidant capacity of cells, we found no effects of this substance on fetal membranes. This may help health care providers make better recommendations about the consumption of one of the worlds’ most popular drinks. In summary, we conclude that up regulation Nrf2 through nutraceutical supplementation may be an ideal and cost-effective approach to reduce the risk of both OS and inflammation-induced adverse pregnancy outcomes.

## 4. Materials and Methods

### 4.1. IRB Ethics Committee Approval Statement

Placentas from cesarean sections for term normal pregnancies (not in labor) were collected from UTMB’s John Sealy Hospital in Galveston, TX after release for disposal by the attending surgeon. The study team had no access to personal health information and did not interact with the subjects at any time. Therefore, the project was determined not to be human subjects research under UTMB IRB in compliance with CFR 45 Part 46. Guidelines.

### 4.2. Tissue-Culture of Normal Term Fetal Membranes

Placentas obtained from elective repeat Cesarean sections at term (>37 weeks of gestation) prior to the onset of labor (*n* = 6) and taken to the laboratory for immediate processing. The fetal membranes were separated from the remainder of the placenta, and the mid-segment portion of the fetal membranes were chosen to avoid any confounding cell population from the placental and or cervical zones in our study. This segment was cleaned with normal saline, removing all visible blood, blood clots and decidua. Cotton gauze soaked in normal saline (pH 7.4) was used to further clean the membrane. Sections were taken from the cleaned fetal membrane for further analysis. Fetal membranes were cut into 6 mm-circles using a biopsy punch and placed in a tissue culture system [73,74]. Tissue biopsies were placed in a Falcon cell culture plate containing 400 µl Dulbecco’s Modified Eagle’s Medium: F12 Ham’s mixture. Media contained 15% (*v*/*v*) heat-inactivated fetal bovine serum (FBS), 1% (*v*/*v*) glutamine solution, 1% (*v*) penicillin/streptomycin solution and 1µl/mL amphotericin B. Cultures were incubated at 37 °C, 5% CO_2_ for 48 h.

### 4.3. Water-Soluble Cigarette Smoke Extract (CSE) Preparation and Stimulation of the Fetal Membranes with CSE and LPS

After a preincubation period of 48 h at 37 °C in an atmosphere of 5% CO_2_, membranes were CSE-stimulated for 6 h. CSE were prepared freshly by bubbling smoke that was drawn from a single lit commercial cigarette that represented high tar through 25 mL of tissue culture medium (DMEM: F12 Ham mixture with antimicrobial agents) [33,34,35]. A 1:10 dilution of CSE media was used for experiments. Additional tissue samples were also treated with LPS (100 ng/mL) for 6 h. Tissue samples from CSE-stimulated cultures and unstimulated control, as well as the media were collected, frozen, and stored at −80°C until processing.

### 4.4. Treatment with SFN and TRN

Fetal membranes were treated with SFN alone (10 µM/mL) (Enzo #ALX-350-230-M010 Enzo Life Sciences, Inc., Farmingdale, NY, USA) or co-treated with SFN along with CSE and LPS for 6 h to document changes associated with Nrf2 and related markers (PPARγ and (HO-1). For TRN treatment, 100mM stock of TRN was prepared by combining 100mg Trigonelline with 729 μL DMSO then diluting 10 μL of this solution in 90 uL media. Fetal membrane explants were exposed to final concentrations of 10 μM of TRN alone or in combination with CSE or 100 ng/mL LPS for 6 h.

### 4.5. Western Blot Analysis for Nrf2 and PPARγ

Protein from nuclear fractions was used for expression characteristics of NRF2, and cytosolic fractions were used for PPARγ by western blot (WB). Nuclear and cytosolic extractions from explants were performed using the kit instructions (NE-PER cat #78833 from ThermoFisher Scientific, Waltham, MA, USA). Briefly, explants were homogenized using a Dounce homogenizer (Fisher Scientific, Atlanta, GA, USA) in the provided CER I buffer containing freshly added protease inhibitors. The homogenates were then vortexed and incubated on ice for 10 min. Cold CER II buffer was then added to the tube, and the homogenates were vortexed for 5 s and incubated on ice for 1 min followed by centrifugation at 16,000× *g* for 5 min. The supernatant containing the cytoplasmic extracts were removed and stored on ice. The pellets containing the nuclei were resuspended in cold NER buffer containing freshly added protease inhibitors, and vortex vigorously on and off for 40 min. The tubes were then centrifuged at 16,000× *g* for 10 min, and the supernatants containing the nuclear fraction were transferred into new tubes and kept on ice. Protein concentrations were estimated in both cytosolic and nuclear fractions (BCA Protein Assay Kit, Thermo Scientific, Waltham, MA, USA) and 20ug of protein was used for each sample to run the western gels as described previously [32]. Immunoreactive proteins were visualized using chemiluminescence reagents ECL WB detection system (Amersham Piscataway, NJ, USA). The anti-human and anti-mouse antibodies used for WBs were as follows: Nrf2 (1:1000, ab62352, Abcam, Cambridge, UK), PPARγ (1:1000,ab19481, Abcam, Cambridge, UK), nuclear protein Histone (D1H2 1:1000, Rabbit mAb, Cell Signaling Technology Danvers, MA, USA) and β-actin (1:15,000, Sigma- Aldrich, St. Louis, MO, USA). The relative levels of the proteins in the specific bands were normalized with either Histone (for Nrf2) or β-actin (for PPARγ) expressions in the same samples, and expressions were densitometrically determined using the Bio-Rad-Image Lab software (version 6.0, Bio-Rad, Hercules, CA, USA).

### 4.6. HO-1 ELISA

Tissue concentration of HO-1 was quantitated using ELISA reagents purchased from Enzo life sciences (ADI-EKS-800 Enzo Life Sciences Farmingdale, NY, NY, USA). Explants were homogenized using the extraction reagent provided with the kit. Protein determination was performed using the standard BCA method. Standards and samples were prepared according to the kit protocol and incubated on the immunoassay plate for 30 min. Wells were then washed six times, and anti-human HO-1 antibody was added to each well, and the plate was further incubated for 1 h. The plate was again washed, and the included anti-rabbit IgG: HRP conjugate was added to each well, and the plate was incubated for 30 min. The plate was washed and TMB substrate was added for color development. The reaction was stopped after 10 min, and absorbance was read at 450nm. A standard curve was used to determine the HO-1 concentration in the samples. The results are documented in ng/mL. The sensitivity of the kit has been determined to be 0.78 ng/mL. The intra-assay coefficient of variation has been determined to be <10%. The inter-assay coefficient of variation has been determined to be <10%. This kit is specific for human HO-1 and does not cross-react with human HO-2 or HO-3.

### 4.7. Luminex Assay to Determine Cytokine Concentration in Culture Supernatants

Culture media samples collected after treatments were assayed for IL-6 using MILLIPLEX Human Cytokine assay (Millipore, Burlington, MA, USA), following the manufacturer’s protocol. Standard curves were developed using duplicate samples of known-quantity recombinant proteins that were provided by the manufacturer. Sample concentrations were determined by relating the absorbance of the samples to the standard curve using linear regression analysis. Data are adjusted to total protein concentrations.

### 4.8. Statistical Analysis

Data were normalized to each subject, and potential differences between treatments were analyzed using ANOVA followed by Tukey’s post-test using GraphPad (Prism 8, San Diego, CA, USA). Results where *p* ≤ 0.05 were considered significant and are presented as mean ± SEM. Data will be made available upon request.
